# Perceived Neighborhood Safety and Active Transportation in Adults from Eight Latin American Countries

**DOI:** 10.3390/ijerph191912811

**Published:** 2022-10-06

**Authors:** Antonio Castillo-Paredes, Beatriz Iglésias, Claudio Farías-Valenzuela, Irina Kovalskys, Georgina Gómez, Attilio Rigotti, Lilia Yadira Cortés, Martha Cecilia Yépez García, Rossina G. Pareja, Marianella Herrera-Cuenca, Mauro Fisberg, Clemens Drenowatz, Paloma Ferrero-Hernández, Gerson Ferrari

**Affiliations:** 1 Grupo AFySE, Investigación en Actividad Física y Salud Escolar, Escuela de Pedagogía en Educación Física, Facultad de Educación, Universidad de Las Américas, Santiago 8370040, Chile; 2 Faculdade de Motricidade Humana, Universidade de Lisboa, 1499-002 Lisbon, Portugal; 3 Sciences of Physical Activity, Sports and Health School, University of Santiago of Chile (USACH), Santiago 9170022, Chile; 4 Faculty of Medical Sciences, Pontificia Universidad Católica Argentina, Buenos Aires C1107, Argentina; 5 Department of Biochemistry, School of Medicine, Universidad de Costa Rica, San José 11501-2060, Costa Rica; 6 Department of Nutrition, Diabetes and Metabolism, School of Medicine, Pontificia Universidad Católica, Santiago 8330024, Chile; 7 Department of Nutrition and Biochemistry, Pontificia Universidad Javeriana, Bogotá 110231, Colombia; 8 Colegio de Ciencias de la Salud, Universidad San Francisco de Quito, Quito 17-1200-841, Ecuador; 9 Instituto de Investigación Nutricional, Lima 15026, Peru; 10 Centro de Estudios del Desarrollo, Universidad Central de Venezuela (CENDES-UCV)/Fundación Bengoa, Caracas 1053, Venezuela; 11 Centro de Excelencia em Nutrição e Dificuldades Alimentaes (CENDA), Instituto Pensi, Fundação José Luiz Egydio Setubal, Hospital Infantil Sabará, São Paulo 01228-200, Brazil; 12 Departamento de Pediatria, Universidade Federal de São Paulo, São Paulo 04023-061, Brazil; 13 Division of Sport, Physical Activity and Health, University of Education Upper Austria, 4020 Linz, Austria; 14 Faculty of Education and Culture, SEK University, Santiago 7520317, Chile; 15 Faculty of Health Sciences, Universidad Autónoma de Chile, Providencia, Santiago 7500912, Chile

**Keywords:** active transportation, active commuting, barriers, Latin America

## Abstract

Neighborhood built environment is associated with domain-specific physical activity. However, few studies with representative samples have examined the association between perceived neighborhood safety indicators and domain-specific active transportation in Latin America. This study aimed to examine the associations of perceived neighborhood safety with domain-specific active transportation in adults from eight Latin American countries. Data were obtained from the Latin American Study of Nutrition and Health (*n* = 8547, aged 18–65). Active transportation (walking and cycling) was assessed using the long form of the International Physical Activity Questionnaire. Specifically, traffic density and speed as well as street lightening, visibility of residents regarding pedestrians and bicyclists, traffic lights and crosswalks, safety of public spaces during the day and at night, crime rate during the day and at night were used to evaluate perceived neighborhood safety. Slow traffic speeds, unsafe public spaces during the day, and crime during the day were associated with ≥10 min/week vs. <10 min/week of walking. Furthermore, drivers exceeding the speed limit and crime rate during the day were associated with reporting ≥10 min/week vs. <10 min/week of cycling. These results indicate a stronger association of the perceived neighborhood safety with walking compared to cycling.

## 1. Introduction

The health benefits of physical activity are well established. They include a lower prevalence of cardiovascular disease, blood pressure, obesity, and mortality [1,2,3,4]. In addition, engagement in physical activity is associated with a decrease in depression, anxiety, stress as well as enhanced cognitive performance and general well-being [5,6,7,8,9]. To obtain these physical and mental health benefits, adults should perform at least 150 to 300 min of moderate physical activity intensity or a minimum of 75 to 150 min of vigorous physical activity throughout the week [10]. The prevalence of physical inactivity (39.1%) in Latin America is the highest reported worldwide [11]. A way to increase physical activity levels in adults could be active transportation (AT), which allows for a reduction in the incidence of non-communicable diseases and all-cause mortality [12].

AT is related to the mode of traveling from one place to another, whether riding a bicycle, walking, or using non-motorized vehicles [13,14,15]. AT has been associated with improved cardiometabolic health and reduced cardiovascular and all-cause mortality [16,17]. Active movement, therefore, could be a viable strategy to increase physical activity, as some movement is better than none [10]. However, psychosocial or environmental barriers have been associated with AT [18,19,20]. A better understanding of barriers and motivators could help with the design of future programs and interventions aimed at the promotion of physical activity at the population level [21].

In urban areas, sports infrastructure or playgrounds, which are located within a 20 min walk have been shown to contribute to moderate-to-vigorous intensity physical activity, as long as they are accessible and considered safe [22]. It has been shown that the built environment in the neighborhood, including available spaces, pedestrian or vehicular traffic and safety, is associated with physical activity during the week [23]. Thus, the exploration of the association of the built environment or environmental attributes could influence decision making about the construction of spaces in a country, city, or neighborhood. In addition, there is a need for spaces for AT in addition to motorized traffic; considering the possibility of harmonious coexistence of pedestrians, cyclists, and motorized vehicles facilitated by spaces or places that may provide a viable contribution to health and the environment [24].

Examining how characteristics of perceived neighborhood safety are associated with domain-specific AT in Latin America may provide useful insights for guiding public policies and strategies for promoting AT in this region [23]. Of particular concern appears to be the perception of safety, as 60% of the Latin American population considers themselves living in an unsafe neighborhood due to interpersonal violence and crime [25]. The literature further reports that 33% of the world’s homicides occur in Latin America, often as part of everyday violence on suburban street corners [26]. Thus, in addition to poor micro infrastructure, the unsafe environment contributes to low levels of AT reported in the region. Safety features were most strongly associated with moderate to high physical activity levels [22]. The highest crime safety was found in Chile (61.7%) and the lowest in Venezuela (24.7%). The countries with the lowest safety at recreational places are Venezuela (15.9%), Argentina (23.2%), and Brazil (25.8%) [22]. In the case of Europe, political documents have established action plans for mobility, prevention of non-communicable diseases, safe walking, and cycling as strategies for moving around in urban environments and thus promoting physical activity [27]. The purpose of this study is to examine the associations of perceived neighborhood safety with domain-specific AT in adults from eight Latin American countries.

## 2. Materials and Methods

### 2.1. Study Design and Sample

The Latin American Nutrition and Health Study (ELANS—https://es.elansstudy.com, accessed on 4 October 2022) is an observational epidemiological study using a common design and comparable methods across eight countries (Argentina, Brazil, Chile, Colombia, Costa Rica, Ecuador, Peru, and Venezuela). The study uses a large representative sample (15–65 years old) from these countries and focuses on urban populations. This study was based on a complex and multistage sampling design, stratified by conglomerates from all the regions of each country, and a random selection of the main cities of each region according to the method of probability proportional to size for the application of a cross-sectional survey by household [28,29].

Data were collected between September 2014 and February 2015. The Western Institutional Review Board approved the ELANS general protocol (no. 20140605) and retrospectively registered at ClinicalTrials.gov (no. NCT02226627) to increase the transparency of the methods and results and avoid criticism about publication bias. Ethical approval was obtained from each local institutional review board. Informed consent was obtained from all participants before data collection.

Within the 92 cities included in the study, a total of 9218 subjects aged 15 to 65 participated in the research. Adolescents aged 15 to 17 years (*n* = 671) were excluded from the present analyses because they may have different lifestyle features [11] compared to those observed in adults. In addition, physical activity guidelines for adolescents differ from those of adults [10]. Thus, the final sample consisted of 8547 adults between 18 and 65 years of age.

### 2.2. Active Transportation

Participants reported their walking and cycling trips using the long version of the International Physical Activity Questionnaire (IPAQ) in Spanish and Portuguese [30]. We adapted the IPAQ using only the questions related to AT. The IPAQ has been validated to assess physical activity in several countries [30,31]. Participants were instructed to report the frequency and duration (bouts of ≥10 min) of AT.

Specifically, the following questions were asked: (a) “During the last 7 days, did you walk or use a bicycle for at least 10 min continuously to get to and from places?” (Yes, No); (b) “During the last 7 days, on how many days did you walk or ride a bicycle for at least 10 min at a time to go from place to place?”; (c) “How much time did you usually spend on one of those days bicycling or walking from place to place?” These questions were asked separately for walking and cycling.

IPAQ data were reported as min/week of walking and cycling for AT. The time (min/week) dedicated to AT was calculated and used in the analysis. AT (walking and cycling) was subsequently dichotomized into <10 min/week vs. ≥10 min/week. This cutpoint (10 min/week or ≥10 min/week) was chosen as the IPAQ assesses activities in 10 min bouts over a period of 7 days for time spent in physical activity in moderate and vigorous intensity as well as walking and cycling [30,31]. For this study, we used walking and cycling separately as modes of AT.

### 2.3. Perceived Neighborhood Safety

To assess the perceived neighborhood safety, the Neighborhood Environment Walkability Scale-Abbreviated (NEWS-A) was used, which has been previously translated into Spanish and adapted for use in Latin America [23,32]. The reliability and validity of the NEWS-A have been assessed with all included scales [33,34].

The questions selected for this study were analyzed separately. For each question, the answers were: (a) totally disagree; (b) in disagreement; (c) in agreement, and (d) agree, which were subsequently dichotomized into disagree and agree (Table 1).

### 2.4. Sociodemographic Variables

The sociodemographic variables included were country, sex (men and women), age, educational level (none/basic education, partial or complete higher education, and university graduate or higher), and ethnicity (mixed/Caucasian, black, white, and others), which were collected using standard questionnaires. Due to differences in the classification systems across countries, we used three categories based on the national statistics of each country and included equivalent characteristics for all countries. Socioeconomic level data was divided into three strata (low, medium, high) based on the national indexes used by each country. Details on data collection for these variables have been published elsewhere [32,35].

### 2.5. Statistical Analysis

The Kolmogorov–Smirnov test and histograms were used to check data normality distribution. Descriptive statistics included absolute and relative frequencies, mean, standard deviation (SD), and median (25th–75th percentile).

Because the walking and cycling variables were positively skewed and had a large number of zeros, two different regression models were used to estimate the associations of each perceived neighborhood safety indicator with AT (walking and cycling separately): a logistic regression model (odds ratio: OR; confidence interval 95%: 95%CI) with a binary dependent variable (0 = “<10 min/week”, 1 = “≥10 min/week”) followed by a linear model with the min/week of AT as the dependent variable. The linear regression model (β; 95%CI) was estimated using data from the respondents who reported ≥10 min of AT per week. Due to the non-normality of data, the minutes of AT were log-transformed for the linear models, and the unstandardized coefficient values were back-transformed into min/week. Both models were adjusted for country, sex, age, socioeconomic level, educational level, and ethnicity. We present the overall (i.e., pooled) results. All analyses were performed using the SPSS V27 software (SPSS Inc., IBM Corp., Armonk, NY, USA).

## 3. Results

### 3.1. Descriptive Data

The descriptive characteristics of participants (*n* = 8547; 47.0% women; 18–65 years [mean age 37.4 years]) are shown in Table 2, stratified by AT. Overall, 52.0% and 59.1% were classified as having low socioeconomic and education level, respectively, and 48.3% identified as mixed/Caucasian. Of the total sample, 76.2% reported walking ≥10 min/week. However, only 9.7% used the bicycle for at least 10 min/week. Median walking and cycling AT were 70.0 (25th–75th: 10.0–175.0) and 0.0 (25th–75th: 0.0–0.0) min/week, respectively. The sociodemographic characteristics and AT by country are shown in Table S1.

Table 3 shows that almost two-thirds (63.4%) agree with the statement that drivers exceed the speed limit, 68.9% report unsafe public space at night and 68.0% report unsafe crime rate at night. On the other hand, 69.9% agree that streets are well lit, and 74.4% indicate that residents can see pedestrians and bicyclists. The perceived neighborhood safety indicators by country are shown in Table S2.

### 3.2. Associations between Safety over Time and Active Transportation

The logistic regression model showed that the odds of reporting ≥10 min/week of walking were higher in participants who agreed with slow traffic speeds (OR: 1.85; 95%CI: 1.65;2.05). In turn, agreement with the statement of unsafe public space during the day (OR: 0.75; 95%CI: 0.64;0.86) and unsafe crime rate during the day (OR:0.66; 95%CI: 0.51;0.81) reduced the odds of reporting ≥10 min/week of walking. Agreement with the statements drivers exceed the speed limit (OR: 0.81; 95%CI: 0.69;0.95) and unsafe crime rate during the day (OR: 0.84; 95%CI: 0.72;0.96) were also associated with lower odds of reporting ≥10 min/week of cycling (Table 4).

### 3.3. Analysis of Reported Walking and Bicycle Use

In Table 5, the linear regression models showed a negative association between unsafe crime rate during the day (β: −12.33; 95%CI: −22.93; −1.73) and walking. No linear associations were reported for perceived neighborhood safety and cycling.

## 4. Discussion

This study examined the associations between perceived neighborhood safety and domain-specific AT in adults from Latin American countries. Perception of slow traffic speeds was associated with higher odds of ≥10 min/week vs. <10 min/week of walking. On the other hand, unsafe public space during the day and unsafe crime rate during the day reduced the odds of reporting ≥10 min/week of walking. Furthermore, drivers exceeding the speed limit and unsafe crime rate during the day were associated with lower odds of reporting ≥10 min/week of cycling.

Speeding, lack of safety, lighting, and crime appear to be critical components in AT. In other words, if the user perceives the environment to be unsafe, he is less likely to walk or cycle from one point to another [36]. Accordingly, a European study showed that increased public lighting improves safety, which contributed to an increase in the use of AT [37]. In addition, the International Physical Activity Network (IPEN) study carried out in 12 countries showed that for a positive association between active movement, whether walking or cycling, and the built environment, there must be a positive perception in the variables of land use, access, street connectivity, aesthetics, and safety [38]. In most European countries, the use of AT in the adult population is common when cycling paths and specific pedestrian places are available [39]. Further urban area planning, however, is required to enhance the built environment in order to contribute to a healthy lifestyle that includes AT [40]. In connection, the Positive Health Effects of the Natural Outdoor Environment in Typical Populations in Different Regions in Europe (PHENOTYPE) project indicated that only 19% of active commuters reported being dissatisfied/very dissatisfied with safety in natural environments (green areas and parks, among others) [41].

In the case of Latin America, sociodemographic differences have been associated with the use of AT in the adult population [42]. It has been shown that in rural areas, there is a wide variety of environmental and physical factors that influence AT [43], compared to urban areas where the use of active movement is greater [44]. However, psychological factors also influence the decision to walk or ride a bicycle to travel from one place to another. Specifically, comfort, the built environment, economy (there is no monetary expense in the use of AT), and safety, the latter being the most important factor in women [19], are essential for AT in urban areas. Therefore, a reconfiguration of the available spaces or of the existing infrastructure must be considered for the use of bicycles, as well as the importance of a change in the behavior of the population in general [20].

Although, at the international level, there is evidence of strategies or action plans for the promotion of physical activity [1,2], it is important to consider the fundamental role of public policies of individual countries for the promotion of physical activity, health, transportation, environment, social and road safety. Therefore, it is necessary to educate decision-makers at the community, regional, or country level about the benefits of reducing passive displacement and increasing AT at the personal, community, social, and environmental levels. An increase in AT can positively impact total physical activity and, thus, decrease the risk for non-communicable diseases and improve physical, emotional, social, and environmental health.

Our study has limitations. Despite the large number of participants per country, the sample is not necessarily representative of the respective localities or countries. The security questions also refer to a “perception” that participants have of the environment where they reside and may not reflect reality. Furthermore, the use of IPAQ does not allow for objective determination of the physical activity contribution by the participant. In turn, this work offers an overview of eight Latin American countries through a set of variables, data, and relevant information available to researchers who could enhance the understanding of various aspects that influence AT in Latin American adults. This information could serve as a starting point for the development of future studies; observational or interventional.

## 5. Conclusions

Moving around actively, whether walking or using a bicycle, is associated with the built environment in a person’s place of residence. Variables related to crime, traffic speed, lack of safety in common spaces/green areas, and lighting of public spaces were associated with AT in countries in Latin America.

More studies, however, are needed that examine the association of spaces, places, and the built environment with the regular practice of AT and possible barriers to its use. This could facilitate a modification of the built environment and contribute to a possible increase in physical activity levels in the adult population of Latin America.

## Figures and Tables

**Table 1:** Summary of perceived neighborhood safety.

Questions	Questions Used in the Results	Categories	Analysis
Environmental barriers		Totally disagree In disagreement In agreement Totally agree	Disagree Agree
“There is a lot of traffic on the streets near my neighborhood, which makes it difficult or unpleasant to walk through them”	A lot of traffic		
“The traffic speed on most of the streets near my neighborhood is usually slow (50 km/h or less)”	Slow traffic speeds		
“Most drivers exceed the speed limit when driving through my neighborhood”	Drivers exceed the speed limit		
“The streets of my neighborhood are well lit at night”	Streets are well lit		
“Residents can easily see pedestrians and bicyclists in their homes”	Residents can see pedestrians and bicyclists		
“There are traffic lights and crosswalks on streets in my neighborhood that help pedestrian traffic on busy streets/heavy traffic”	There are traffic lights and crosswalks on streets		
“The parks, public squares, green areas and places of recreation in my neighborhood are unsafe during the day”	Unsafe public space during the day		
“The parks, public squares, green areas and places of recreation in my neighborhood are unsafe at night”	Unsafe public space at night		
Psychosocial barriers of crime			
“There is a high crime rate in my neighborhood”	High crime rate		
“The crime rate in my neighborhood makes it unsafe to walk during the day”	Unsafe crime rate during the day		
“The crime rate in my neighborhood makes it unsafe to walk around at night”	Unsafe crime rate at night		

**Table 2:** Sociodemographic characteristics and active transportation in overall.

Variables	Total	Walking	Cycling
		≥10 Min/Week (%)	≥10 Min/Week (%)
Sample (*n*)	8547	76.2	9.7
Sex (%)			
Men	47.0	73.0	15.0
Women	53.0	79.0	5.0
Socioeconomic level (%)			
Low	52.0	76.6	9.6
Medium	38.5	75.8	9.9
High	9.5	75.6	10.0
Education level (%)			
None/basic education	59.1	76.2	10.7
Partial or complete higher education	30.8	77.6	8.7
University graduate or higher	10.1	71.5	7.1
Ethnicity (%)			
Mixed/Caucasian	48.3	79.4	9.0
Black	6.7	74.2	11.2
White	36.7	72.4	9.8
Others	8.3	77.0	12.3

**Table 3:** Perceived neighborhood safety indicator and active transportation in overall.

Variables	Total	Walking	Cycling
		≥10 Min/Week (%)	≥10 Min/Week (%)
Environmental barriers			
A lot of traffic			
Agreement	55.6	75.3	10.2
Disagreement	44.4	77.5	9.8
Slow traffic speeds			
Agreement	55.4	75.2	10.5
Disagreement	44.6	77.6	9.4
Drivers exceed the speed limit			
Agreement	63.4	75.5	10.6
Disagreement	36.6	77.4	8.5
Streets are well lit			
Agreement	69.9	76.7	10.3
Disagreement	30.1	29.5	9.5
Residents can see pedestrians and bicyclists			
Agreement	74.4	76.5	10.5
Disagreement	25.6	75.7	8.7
There are traffic lights and crosswalks on streets			
Agreement	46.4	75.3	10.5
Disagreement	53.6	77.3	9.4
Unsafe public space during the day			
Agreement	40.3	72.5	10.0
Disagreement	59.7	78.7	10.4
Unsafe public space at night			
Agreement	68.9	75.4	9.9
Disagreement	31.1	78.0	10.4
Psychosocial barriers of crime			
High crime rate			
Agreement	60.2	75.4	9.7
Disagreement	39.8	77.6	10.6
Unsafe crime rate during the day			
Agreement	40.0	72.6	9.7
Disagreement	60.0	78.5	10.1
Unsafe crime rate at night			
Agreement	68.0	75.8	9.5
Disagreement	32.0	77.2	10.5

**Table 4:** Association (OR; 95%CI) between perceived neighborhood safety and active transportation (0 = <10 min/week, 1 = ≥10 min/week).

Neighborhood Safety	Walking *		Cycling *	
	OR (95%CI)	*p*	OR (95%CI)	*p*
Environmental barriers				
A lot of traffic				
Agreement	1		1	
Disagreement	1.02 (0.92;1.14)	0.606	0.94 (0.80;1.09)	0.441
Slow traffic speeds				
Agreement	1		1	
Disagreement	1.85 (1.65;2.05)	0.005	1.00 (0.86;1.17)	0.943
Drivers exceed the speed limit				
Agreement	1		1	
Disagreement	1.10 (0.99;1.23)	0.068	0.81 (0.69;0.95)	0.012
Streets are well lit				
Agreement	1		1	
Disagreement	1.05 (0.93;1.17)	0.412	1.09 (0.92;1.29)	0.314
Residents can see pedestrians and bicyclists				
Agreement	1		1	
Disagreement	0.97 (0.86;1.10)	0.723	0.88 (0.73;1.06)	0.185
There are traffic lights and crosswalks on streets				
Agreement	1		1	
Disagreement	1.05 (0.94;1.17)	0.350	0.98 (0.84;1.15)	0.864
Unsafe public space during the day				
Disagreement	1		1	
Agreement	0.75 (0.64;0.86)	<0.001	0.94 (0.80;1.11)	0.51
Unsafe public space at night				
Agreement	1		1	
Disagreement	1.03 (0.91;1.15)	0.628	0.97 (0.82;1.14)	0.737
Psychosocial barriers of crime				
High crime rate				
Agreement	1		1	
Disagreement	1.04 (0.93;1.16)	0.490	0.97 (0.83;1.13)	0.723
Unsafe crime rate during the day				
Agreement	1		1	
Disagreement	0.66 (0.51;0.81)	<0.001	0.84 (0.72;0.96)	0.041
Unsafe crime rate at night				
Agreement	1		1	
Disagreement	0.96 (0.86;1.08)	0.557	0.95 (0.81;1.12)	0.571

* Model adjusted for country, sex, age, socioeconomic level, educational level, and ethnicity. OR: odds ration; CI: confidence interval.

**Table 5:** Association (β; 95%CI) between perceived neighborhood safety and active transportation.

Neighborhood Safety	Walking Participants ≥ 10 Min/Week *		Cycling Participants ≥ 10 Min/Week *	
	β (95%CI)	*p*	β (95%CI)	*p*
Environmental barriers				
A lot of traffic				
Disagreement	1		1	
Agreement	−3.23 (−13.63;7.16)	0.542	2.63 (−2.55;7.82)	0.32
Slow traffic speeds				
Disagreement	1		1	
Agreement	−6.75 (−17.09;3.64)	0.204	1.63 (−3.54;6.81)	0.536
Drivers exceed the speed limit				
Disagreement	1		1	
Agreement	−1.18 (−11.91;9.53)	0.828	−2.70 (−8.05;2.64)	0.321
Streets are well lit				
Disagreement	1		1	
Agreement	1.79 (−9.50;13.09)	0.756	−0.74 (−6.37;4.88)	0.796
Residents can see pedestrians and bicyclists				
Disagreement	1		1	
Agreement	2.84 (−8.96;14.64)	0.637	3.40 (−2.47;9.28)	0.257
There are traffic lights and crosswalks on streets				
Disagreement	1		1	
Agreement	10.12 (−0.51;20.77)	0.062	3.44 (−1.86;8.76)	0.204
Unsafe public space during the day				
Disagreement	1		1	
Agreement	−8.54 (−19.13;2.04)	0.114	−0.70 (−5.98;4.58)	0.794
Unsafe public space at night				
Disagreement	1		1	
Agreement	−7.67 (−18.88–3.53)	0.179	−3.14 (−8.73;2.45)	0.271
Psychosocial barriers of crime				
High crime rate				
Disagreement	1		1	
Agreement	−2.64 (−13.27;7.98)	0.626	−2.57 (−7.88;2.73)	0.341
Unsafe crime rate during the day				
Disagreement	1		1	
Agreement	−12.33 (−22.93;−1.73)	0.023	3.10 (−2.17;8.39)	0.249
Unsafe crime rate at night				
Disagreement	1		1	
Agreement	−0.677 (−11.807;10.452)	0.905	−0.12 (−5.68;5.42)	0.964

* Model adjusted for country, sex, age, socioeconomic level, educational level, and ethnicity. CI: confidence interval.

## Data Availability

The study database is not available for general use due to the terms of consent/assents signed by the participants. It is suggested to contact the author by correspondence for more information or availability of information.

## Supplementary Materials

The following supporting information can be downloaded at: https://www.mdpi.com/article/10.3390/ijerph191912811/s1, Table S1: Sociodemographic characteristics and active transportation by country; Table S2: Characterization of the sample by questions about neighborhood safety by country.
