# Supplementary material for: Perceived Neighborhood Safety and Active Transportation in Adults from Eight Latin American Countries
Source: Int J Environ Res Public Health. 2022 Oct 6;19(19):12811. doi: 10.3390/ijerph191912811 (PMC9566435; doi:10.3390/ijerph191912811)
Supplement: Supplementary file 1 [file ijerph-19-12811-s001.zip › ijerph-1882295-supplementary.pdf]

Table S1. Sociodemographic characteristics and active transportation by country.

Variables	Argentina	Brazil	Chile	Colombia	Costa Rica	Ecuador	Peru	Venezuela
Sample (n)	1177	1872	811	1154	728	737	1018	1050
Age (Mean [SD])	38.3 (13.1)	37.9 (13.1)	38.1 (13.3)	38.3 (14.0)	37 (13.2)	35.8 (13.4)	35.9 (12.9)	36.5 (13.3)
Sex (%)								
Men	43.7	45.9	47.5	48.3	49.0	49.3	46.5	48.4
Women	56.3	54.1	52.5	51.7	51.0	50.7	53.5	51.6
Socioeconomic level (%)								
Low	47.2	46.4	46.7	62.8	32.7	50.5	48.1	77.7
Medium	47.5	45.2	44.3	31.7	53.6	36.6	31.5	17.1
High	5.3	8.4	9.0	5.5	13.7	12.9	20.4	5.2
Education level (%)								
None/Basic education	73.8	45.4	62.6	63.5	79.9	81.5	19.4	67.5
Partial or complete higher education	21.7	45.6	25.3	24.7	13.7	11.4	70.0	13.3
University graduate or higher	4.5	9.0	12.1	11.8	6.4	7.1	10.6	19.2
Ethnicity (%)								
Mixed/Caucasian	25.3	18.8	56.9	61.2	34.7	87.9	89.4	47.2
Black	0.0	20.6	0.0	8.2	2.1	3.0	0.7	4.1
White	71.6	41.5	38.6	25.0	50.4	4.6	8.9	42.2
Others	3.1	19.1	4.5	5.6	12.8	4.5	1.0	6.5
Walking								
≥10 min/week (%)								
Yes	69.6	73.1	75.1	80.1	83.1	89.8	85.1	62.6
No	30.4	26.9	24.9	19.9	16.9	10.2	14.9	37.4
≥10 min/week (Total min)								
Mean (SD)	152.8 (250.2)	128.3 (216.1)	132.5 (204.1)	156.4 (243.2)	201.2 (288.3)	188.7 (250.2)	160.2 (219.7)	103.3 (192.0)
Median (P25-P75)	60.0 (0.0 - 180.0)	60.0 (0.0 - 140.0)	70.0 (10.0 - 150.0)	76.5 (20.0 - 180.0)	100.0 (30.0 - 240.0)	105.0 (60.0 - 210.0)	90.0 (36.0 - 200.0)	45.0 (0.0 - 120.0)
Cycling								
≥10 min/week (%)								
Yes	10.8	11.4	12.9	9.9	15.2	8.5	6.7	2.7
No	89.2	88.6	87.0	90.1	84.7	91.5	93.3	97.3
≥10 min/week (Total min)								
Mean (SD)	26.7 (129.7)	22.4 (113.3)	36.4 (173.7)	28.7 (135.9)	33.9 (144.1)	12.8 (72.9)	8.5 (58.0)	6.0 (73.4)
Median (P25-P75)	0.0 (0.0 - 0.0)	0.0 (0.0 - 0.0)	0.0 (0.0 - 0.0)	0.0 (0.0 - 0.0)	0.0 (0.0 - 0.0)	0.0 (0.0 - 0.0)	0.0 (0.0 - 0.0)	0.0 (0.0 - 0.0)

SD: standard deviation; P: percentile.

Table S2. Characterization of the sample by questions about neighborhood safety by country.

Safety (%)	Argentina	Brazil	Chile	Colombia	Costa Rica	Ecuador	Perú	Venezuela
Environmental barriers								
A lot of traffic								
Agreement	61.6	62	59.8	52.9	56.2	49.8	38.5	57.4
Disagreement	38.4	38	40.2	47.1	43.8	50.2	61.5	42.6
Slow traffic speeds								
Agreement	47.8	63.6	56	55.5	51.4	56.9	56	50
Disagreement	52.2	36.4	44	44.5	48.6	43.1	44	50
Drivers exceed the speed limit								
Agreement	66.9	71.3	63.4	58.8	65	62.4	53.7	59.2
Disagreement	33.1	28.7	36.6	41.2	35	37.6	46.3	40.8
Streets are well lit								
Agreement	65.6	68.3	81.3	77.1	78.4	74.1	73.4	49
Disagreement	34.4	31.7	18.7	22.9	21.6	25.9	26.6	51
Residents can see pedestrians and bicyclists								
Agreement	77.4	71.6	74.4	80.2	73.9	79.6	73	67.5
Disagreement	22.6	28.4	25.6	19.8	26.1	20.4	27	32.5
There are traffic lights and crosswalks on streets								
Agreement	51.3	55.6	70.5	38.3	43.3	43	31.7	33.2
Disagreement	48.7	44.4	29.5	61.7	56.7	57	68.3	66.8
Unsafe public space during the day								
Agreement	48.9	48.6	21.6	35.3	34.6	32.7	23.5	61.7
Disagreement	51.1	51.4	78.4	64.7	65.4	67.3	76.5	38.3
Unsafe public space at night								
Agreement	76.8	74.5	56.1	59.1	70.1	64.5	58.1	83.7
Disagreement	23.2	25.5	43.9	40.9	29.9	35.5	41.9	16.3
Psychosocial barriers of crime								
High crime rate								
Agreement	67.6	67.6	37.5	56.9	56.9	56.3	50	75
Disagreement	32.4	32.4	62.5	43.1	43.1	43.7	50	25
Unsafe crime rate during the day								
Agreement	49.5	48.9	18.1	37	33.2	30.7	23.4	61

Disagreement	50.5	51.1	81.9	63	66.8	69.3	76.6	39
Unsafe crime rate at night								
Agreement	75.8	73.8	51.8	59.1	67.9	66.1	58.2	82.6
Disagreement	24.2	26.2	48.2	40.9	32.1	33.9	41.8	17.4
